# A Pilot Study on the Proteomics Profile of Serum Exosome-Enriched Extracellular Vesicles from Normal versus Individuals with Obesity-Related Insulin Resistance

**DOI:** 10.3390/biomedicines12040799

**Published:** 2024-04-03

**Authors:** Viswanathan Saraswathi, Weilun Ai, Vikas Kumar, Kanika Sharma, Thiyagarajan Gopal, Narendra Kumar, Harmeet Malhi, Tejasav Sehrawat, Cyrus V. Desouza

**Affiliations:** 1 VA Nebraska-Western Iowa Health Care System, Omaha, NE 68105, USA; s.viswanathan@unmc.edu (V.S.); weilun.ai@unmc.edu (W.A.); thiyagarajan.clatr@sathyabama.ac.in (T.G.); narendra@dr.du.ac.in (N.K.); 2 Department of Internal Medicine, Division of Diabetes, Endocrinology, and Metabolism, University of Nebraska Medical Center, Omaha, NE 68198, USA; 3 Department of Genetics Cell Biology & Anatomy, University of Nebraska Medical Center, Omaha, NE 68198, USA; vikas.kumar@unmc.edu (V.K.); kasharma@unmc.edu (K.S.); 4 Department of Internal Medicine, Division of Gastroenterology and Hepatology, Mayo Clinic College of Medicine, Rochester, MN 55905, USAtejasav@gmail.com (T.S.)

**Keywords:** obesity, insulin resistance, diabetes, proteomics

## Abstract

Objective: Circulating exosome-enriched extracellular vesicles (EVs) have drawn considerable importance in obesity-related insulin-resistance (IR). We sought to compare the proteomics profile of serum exosomes from normal individuals and those with obesity and IR. Methods: We isolated serum exosomes from male subjects with obesity and insulin resistance (Ob-IR, HOMA-IR > 2.0) and lean/overweight insulin-sensitive (Normal (N), HOMA-IR < 2.0) individuals. The differential protein expression between the two groups was detected by a label-free quantitative mass spectrometry analysis followed by GO annotation and ingenuity pathway analysis (IPA). Results: We identified 23 upregulated and 46 downregulated proteins between Ob-IR and N groups. Some of these proteins are involved in altering insulin signaling (VPS13C, TBC1D32, TTR, and ADIPOQ), inflammation (NFκB and CRP), and B-cell proliferation/activation (IGLV4-69, IGKV1D-13, and IGHV4-28). GO analysis revealed that the differentially expressed proteins (DEPs) are mainly involved in regulating immune cell activation and are located in extracellular space. IPA analysis showed that top molecules mediating IR, inflammation and B-cell activation were upregulated in Ob-IR subjects compared to N subjects. Conclusions: Serum exosomal proteins can be used as biomarkers to identify the future risk of diabetes and a therapeutic target to prevent or slow down the progression of diabetes in high-risk individuals.

## 1. Introduction

The ever-rising worldwide prevalence of obesity and endocrine co-morbidities like insulin resistance (IR) makes it a public health problem [1,2]. Obesity-related IR has been a great challenge to clinicians and researchers due to the multifactorial nature of its pathogenesis affecting early diagnosis and effective therapeutic interventions [3]. Given that obesity and IR are vastly studied, it is known that multiple integrative approaches are needed to understand their complex biochemical and pathophysiological mechanisms. Obesity-related IR is a major risk factor for the development of type 2 diabetes (T2D), but the underlying mechanisms by which obesity-related IR progresses to T2D remains unclear. Moreover, glucose tolerance tests and hyperinsulinemic and euglycemic clamp studies are needed to confirm IR. However, these tests are not performed routinely to identify individuals with a high risk for developing T2D.

Recently, exosomes or extracellular vesicles (EVs) have been proven to act as paracrine communicators between cells, which has attracted the attention of many scientists. Exosomes play important roles in cell–cell communication and participate in several pathophysiological processes [4]. Exosomes are small extracellular membrane vesicles that are secreted by cells and contain proteins, lipids, and nucleic acids that can be taken up by other cells. They have been widely studied for their role as biomarkers or in the pathogenesis of various diseases.

Regarding obesity and its co-morbidities, several lines of evidence suggest that exosomal cargos, in particular, microRNAs, are altered in the plasma and/or adipose tissue (AT) of humans and are shown to participate in the development of IR (reviewed in [5]). However, only a few studies have shown that the protein cargo of exosomes are also altered in obesity. For example, Ge et al. have shown that circulating exosomes from non-diabetic individuals with obesity showed lower levels of omentin 1 which has been shown to be a mediator linking IR with β-cell function [6]. Another study has shown that transforming growth factor beta 1 (TGFBI) in circulating EVs may facilitate monitoring the T2D status in obese patients, and EV-mimecan (*aka* osteoglycin) may be useful to track visceral obesity [7]. Proteomics analysis of exosomes from mouse plasma has revealed an upregulation of two proteins (immunoglobulins) and downregulation of 10 proteins (14-3-3 protein isoforms, proteasome subunits, and others) in mice with diet-induced obesity [8]. Proteomics analysis has also been performed in adipocytes of human subjects which revealed that changes in proteins related to lipid metabolism and unfolded protein response discriminate insulin-resistant and insulin-sensitive individuals with unequal adiposity [9]. In addition to serving as a biomarker, the exosomal proteomics profile may also reveal the therapy response. For example, in a recent study, plasma exosomal proteomics cargo has been shown to alter in response to lifestyle interventions in adolescents with hepatic steatosis [10]. 

Research on exosome proteomes in obesity-related IR is still in its early stages, and more studies are needed to fully understand the role of exosomal cargo proteins as biomarkers or therapeutic targets to prevent/manage T2D in humans. Our study aims to investigate the differential expression of serum exosomal proteins in normal subjects (N) and individuals with obesity and IR. Using the label-free quantitative mass spectrometry (MS)-based proteomic approach, we analyzed the differential expression of serum exosomal proteins in N and Ob-IR groups. 

## 2. Methodology

### 2.1. Study Design and Subjects 

In this study, serum samples from 4 male subjects with obesity and IR and 4 lean/overweight insulin-sensitive men were used for exosome isolation. All blood samples for serum isolation were obtained while subjects were in a fasting state. The following inclusion and exclusion criteria were applied while choosing the subjects for the study. *Inclusion criteria.* (1) Age 19 to 75; (2) lean/Over-weight insulin sensitive: BMI between 20–29.9 kg/m^2^ and no insulin resistance (HOMA-IR < 2.0); obesity and insulin resistance: BMI 30–55 kg/m^2^ and with HOMA-IR ≥ 2; and (3) subjects should be on a stable dose of any medications for at least two months. *Exclusion Criteria.* (1) Patients currently taking NSAIDs more than 3/week on a prescription basis or taking a daily dose of NSAID; (2) history of diabetes and patients taking diabetes medications as these drugs may alter adipose tissue metabolic functions; (3) history of uncontrolled hypertension defined as >160 systolic and 95 diastolic on medication; (4) history of renal disease with GFR < 60; (5) history of hepatic failure or AST/ALT > three times the normal range; (6) patients with active cancer within the last 2 years except for skin cancers; (7) patients with acute illness needing hospitalization within the last 2 months; (8) patients with acute inflammation; (9) patients with cardiovascular events such as myocardial infarction, stroke, amputation, unstable angina within the last six months; (10) pregnancy; and (11) presence of psychosis, suicidal ideations, untreated major depression, dementia and history of stimulant dependence/substance abuse. The study was approved by the Institutional Review Board at the VA Nebraska-Western Iowa Health Care System (NWIHCS). All participants gave informed consent.

### 2.2. Isolation of Exosomes 

The ultracentrifugation (UC) method has been widely used for proteomics analysis of exosomes because it provides high-purity exosomes. We isolated exosomes using the UC method as reported earlier with slight modifications [11]. Briefly, 1.5 mL of the serum samples was centrifuged at 2000× *g* for 15 min at 4 °C. Supernatant was diluted 1:3 volumes in sterile phosphate-buffered saline (PBS) and centrifuged at 2000× *g* for 30 min at 4 °C. The supernatants were centrifuged at 10,000× *g* for 45 min at 4 °C to remove larger vesicles. Prior to ultrafiltration, samples were filtered through a 0.45 μm filter. The supernatant was centrifuged at 100,000× *g* for 2 h at 4 °C using a Beckman ultracentrifuge. The pellet was resuspended in PBS, filtered again (0.45 μm) and centrifuged at 100,000× *g* for 2 h at 4 °C. The pellet (containing exosomes) was resuspended in PBS, aliquoted and kept at −80 °C until further use.

### 2.3. Nanoparticle Tracking Analysis

An aliquot of freshly isolated exosomes or samples stored at 4 °C overnight was used for nanoparticle tracking analysis (NTA) to determine the size distribution of exosomes using the NanoSight NS300 (Malvern, Malvern, UK) with a 405 nm laser instrument. Samples were analyzed using the basic control settings: infusion Rate, 20 µL/min; detection threshold, 5; and camera level, 10–15. Data were analyzed using the NTA 3.0 software.

### 2.4. Electron Microscopic Imaging of Exosomes 

Morphologies of exosomes were determined using the transmission electron microscopy (TEM) Electron microscopic studies were carried out at the University of Nebraska Medical Center’s electron microscopy core facility using FEI Tecnai G2 Spirit transmission electron microscope (Field Electron and Ion Company, Hillsboro, OR, USA) operated at 80 kv.

### 2.5. Label-Free Quantitative Mass Spectrometry and Data Analysis 

Serum exosomes were lysed using the RIPA buffer (J62524.AE, Thermo Fisher Scientific, Waltham, MA, USA) containing protease and a phosphatase inhibitor cocktail (78444, Thermo Fisher Scientific). For proteomics analysis, 50 µg of protein per sample from four biological replicates per group was taken and the detergent was removed by chloroform/methanol extraction. The protein pellet was re-suspended in 100 mM ammonium bicarbonate and digested with MS-grade Pierce trypsin (Thermo Fisher scientific, Waltham, MA, USA) overnight at 37 °C following reduction with 10 mM DTT at 56 °C for 30 min and alkylation using 50 mM iodoacetamide at RT for 25 min. Peptides were cleaned with PepClean C18 spin columns (Thermo) and were re-suspended in 2% acetonitrile (ACN) and 0.1% formic acid (FA). Each sample containing 500 ng of protein was loaded onto trap column Acclaim PepMap 100 75 µm × 2 cm C18 LC columns (Thermo Scientific™) at a flow rate of 4 µL/min and then separated with a Thermo RSLC Ultimate 3000 (Thermo Scientific™) on a Thermo Easy-Spray PepMap RSLC C18 75 µm × 50 cm C-18 2 µm column (Thermo Scientific™). A step gradient of 4–25% solvent B (0.1% FA in 80% ACN) from 10 to 100 min and 25–45% solvent B for 100 to 130 min at 300 nL/min was used at 50 °C with a 155 min total run time. Eluted peptides were analyzed using a Thermo Orbitrap Exploris 480 (Thermo Scientific™) mass spectrometer in a data-dependent acquisition mode. A survey full scan MS (from *m*/*z* 350–1200) was acquired in the Orbitrap with a resolution of 60,000. The Normalized AGC target for MS1 was set as 300% and the ion filling time as 25 ms. The most intense ions with charge states 2–6 were isolated in 3 s cycle and fragmented using the higher-energy collisional dissociation (HCD) fragmentation method with a 30% normalized collision energy detected at a mass resolution of 15,000 at 200 *m*/*z*. The AGC target for MS/MS was set as 50% and the ion filling time set to auto for 30 s with a 10 ppm mass window. Protein identification was performed by searching MS/MS data against the swiss-prot Homo sapiens protein database downloaded in December 2022 using the in-house PEAKS X + DB search engine. The search was set up for full tryptic peptides with a maximum of two missed cleavage sites. Acetylation of protein N-terminus and oxidized methionine were included as variable modifications and carbamidomethylation of cysteine was set as fixed modification. The precursor mass tolerance threshold was set at 10 ppm and the maximum fragment mass error was 0.02 Da. The significance threshold of the ion score was calculated based on a false discovery rate of ≤1%. Quantitative data analysis was performed using progenesis QI proteomics 4.2 (Nonlinear Dynamics, Waters Corporation, Milford, MA, USA). Proteomics data have been deposited to the MassIVE repository, a member of the ProteomeXchange Consortium (PXD045735).

### 2.6. Western Blot Analysis

Exosome lysates were subjected to SDS-PAGE under reducing and heat-denaturing conditions using Bis-Tris Plus Mini Protein Gels (4–12%, NW04125BOX, Thermo Fisher Scientific) and MOPS SDS Running Buffer (B000102, Thermo Fisher Scientific). Then, the proteins were transferred to the 0.2 μm PVDF membrane (ISEQ00010, Millipore, Burlington, MA, USA) using the transfer buffer (BT0006, Thermo Fisher Scientific) containing 10% methanol. After transferring, the membrane was incubated with 5% fat-free milk powder for 1 h at room temperature. After blocking by 5% fat-free milk powder, membranes were incubated in appropriate primary antibodies overnight at 4 °C to detect target proteins. The primary antibodies against CD9 (10626D, Invitrogen, Waltham, MA, USA), flotillin 1 (18634, Cell Signaling Technology, Danvers, MA, USA), GM130 (12480, Cell Signaling Technology), Calnexin (2433S, Cell Signaling Technology), VPS13C (28676-1-AP, Proteintech, Rosemont, IL, USA), adiponectin (21613-1-AP, Proteintech), and immunoglobulin κ light chain (14678-1-AP), were used for immuno-detection at 1:1000 dilution. After washing three times with TBS buffer containing 0.5% Tween-20 (TBST), membranes were incubated with anti-rabbit (7074, Cell Signaling) or anti-mouse (7076, Cell Signaling) secondary antibody conjugated to HRP-linked antibody at 1:5000 dilution or 1 h at room temperature. Secondary antibody signals were revealed by enhanced chemiluminescence reagent (1705062, Biorad, Hercules, CA, USA). To normalize the protein bands with total protein, exosome samples were run on a gel. The gel was fixed in 50% methanol containing 10% acetic acid for 1 h. Then, the protein gel was subjected to staining with 0.1% Coomassie Brilliant Blue R-250 (1610400, Biorad, Hercules, CA, USA) for 3 h. The gel was then de-stained using 50% methanol plus 10% acetic acid for 2 h.

### 2.7. Statistical Analysis 

Statistical analysis was performed using the one-way ANOVA and the Benjamini-Hochberg (BH) method was used to adjust the *p* values for multiple-testing-caused false discovery rate (FDR). The adjusted *p* ≤ 0.05 was considered significant. Various plots such as PCA, Venn diagram, and volcano plot were generated using Partek Genomics Suite 7.0. GO pathway enrichment was used to analyze the proteins involved in the biological process, cellular component, and molecular function. Western blot data were analyzed by one-tailed Student’s *t*-test. 

## 3. Results

### 3.1. Characteristics of Study Groups 

The anthropometric and clinical characteristics of the study subjects are summarized in Table 1. As expected, the BMI and HOMA-IR used in the selection of the two groups are different between the two groups. In addition, body weight, diastolic blood pressure, glucose, insulin, and alanine aminotransferase (ALT) were significantly higher in Ob-IR versus N subjects. No change in other variables including total cholesterol, triglycerides, LDL cholesterol, and HDL cholesterol were noted.

### 3.2. Isolation and Characterization of Serum Exosomes in Obesity-Related IR

**Figure 1:**
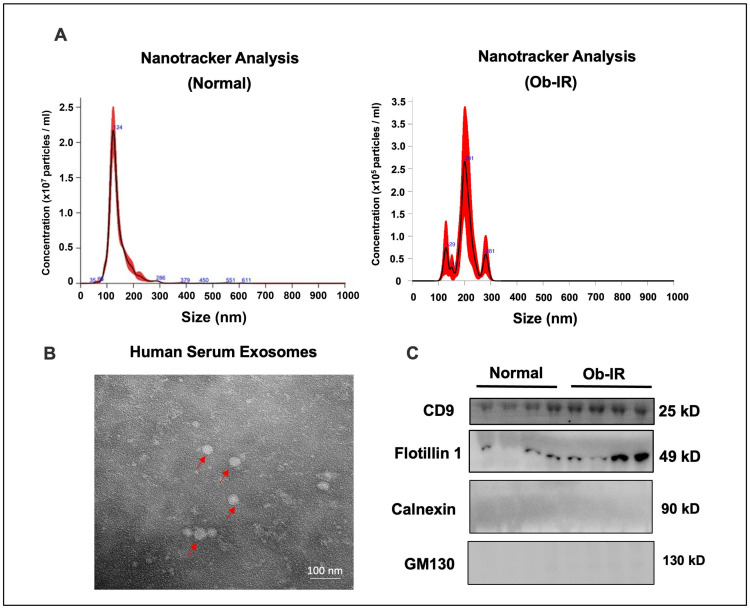
Characterization of exosome-enriched extracellular vesicles (EVs) isolated from human serum. (**A**) Representative histogram of particle concentration and size distribution profile of EVs determined by the nanotracker analysis (NTA). (**B**) Electron microscopic picture of serum exosomes. (**C**) Western blot analysis showing exosome markers.

The exosomes were isolated from the serum samples by ultracentrifugation. NTA was used to measure the size and concentration based on the tracking of Brownian movement. The mean size (196 ± 33.7 and 228 ± 35.7 in N and Ob-IR groups, respectively) and concentration (4.78 × 10^8^ ± 2.66 × 10^8^ and 1.68 × 10^7^ ± 3.91 × 10^6^ in N and Ob-IR groups, respectively) did not vary significantly between groups. A representative NTA plot for each group is shown in Figure 1A. TEM was utilized to further characterize the morphology of the exosomes. The exosomes appeared as round vesicles of heterogenous sizes (Figure 1B). Next, we performed the Western blot analysis to detect exosome markers. The exosome markers including CD9 and flotillin 1 were detected in our samples. On the other hand, calnexin and GM130, a marker of endoplasmic reticulum and Golgi, respectively, were not altered, indicating that our samples mostly contained EVs (Figure 1C). Taken together, these results demonstrated that the exosomes were successfully isolated from the serum with high purity and well-characterized by various methods.

### 3.3. Identification of DEPs between Ob-IR and N Groups

**Figure 2:**
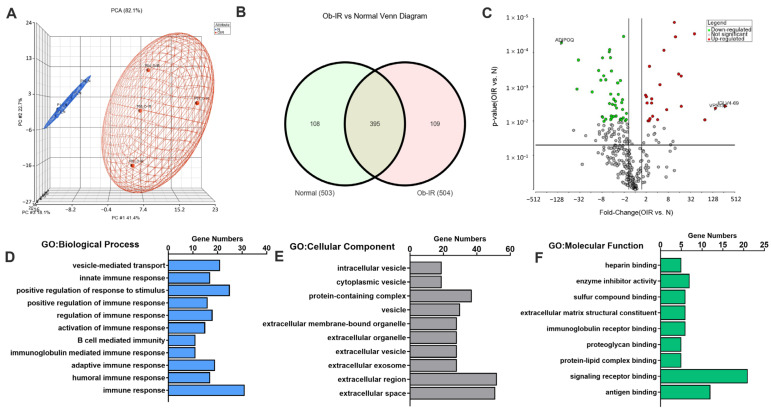
Proteomic analysis of serum-derived exosomes. (**A**) Principle Component Analysis plot. (**B**) The Venn diagram displays the distribution of exosomal proteins between normal subjects (N) and individuals with obesity and insulin resistance (Ob-IR). (**C**) Volcano plot showed the significantly altered proteins between the N and Ob-IR groups. The ratio of expression of these proteins in Ob-IR vs. N are plotted against the *p* value. The red and green dots indicate the up- and downregulated proteins, respectively. The gray dots indicate proteins that had no significant difference. (**D**–**F**) The gene ontology (GO) enrichment analyses of differentially expressed proteins (DEPs). GO enrichment analysis of DEPs in the (**D**) biological process, (**E**) cellular component, and (**F**) the molecular function categories. All significantly enriched GO terms (*p* < 0.05) involving DEPs are displayed.

We next performed the proteomics analysis on the exosome samples. The principal component analysis (PCA) was performed for assessing the quality of data which shows distinct clustering of the proteome between groups (Figure 2A). A total of 503 and 504 proteins were profiled in N and Ob-IR groups, respectively. The Venn diagram shows that 108 proteins are present only in the normal group and 109 proteins are found only in the Ob-IR group whereas 395 proteins are present in both groups (Figure 2B). Only proteins detected in both groups were used for further analysis. We noted that 69 exosomal proteins were differentially expressed (23 upregulated and 46 downregulated) at an adjusted *p* < 0.05. The DEPs are depicted in a volcano plot (Figure 2C). 

### 3.4. GO Enrichment Analysis

All the DEPs were subjected to GO enrichment analysis which shows the enrichment of genes in three categories: (1) biological processes, (2) cellular component, and (3) molecular function (Figure 2D–F). Regarding biological processes, most of the DEPs were enriched in the humoral and adaptive immune response, immune system process, and B-cell-mediated immunity. DEPs enriched in the cellular component were those present in the extracellular region, extracellular space, extracellular vesicle, extracellular exosome, and cell periphery, indicating that the DEPs are present mainly in the exosome fraction. DEPs regulating the molecular functions are mostly enriched in signaling receptor binding, antigen binding, and immunoglobulin receptor binding, suggesting an altered immune response between groups. Together the GO enrichment analysis shows that DEPs are mostly enriched in exosomes and modulate processes regulating the immune response. 

**Figure 3:**
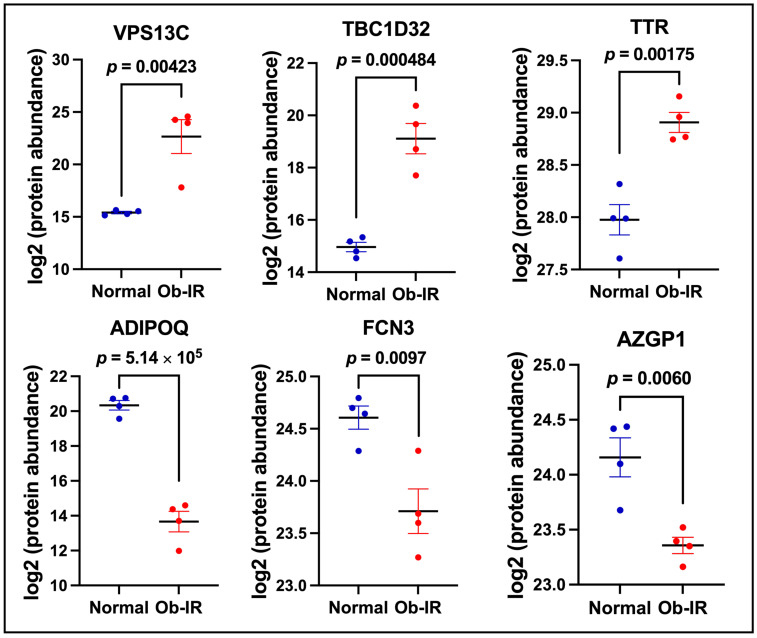
Markers of insulin resistance in exosomes. Proteins altering insulin signaling and/or glucose uptake in exosomes from normal and Ob-IR subjects. Values are mean ± SEM of 4 samples per group.

A detailed list of DEPs with fold changes and *p*-values are shown in Table 2. Regarding specific markers associated with IR and T2D risk, we noted a striking increase in the vacuolar protein-sorting 13 homolog C (VPS13C, 152-fold, *p* = 4.23 × 10^−3^) in Ob-IR subjects compared to N individuals. In addition, the levels of TBC1 Domain Family Member 32 (TBC1D32, 17.75-fold, *p* = 4.84 × 10^−4^) and transthyretin (TTR, 1.9-fold, *p* = 1.75 × 10^−3^) were increased in the Ob-IR group. On the other hand, a profound reduction in proteins improving insulin sensitivity, in particular, adiponectin, an insulin-sensitizing adipokine, was seen in Ob-IR compared to N subjects (ADIPOQ, −101.996-fold, *p* = 5.14 × 10^−5^). Further, we noted a reduction in the levels of ficolin 3 (FCN3, −1.86-fold, *p* = 9.7 × 10^−3^) and zinc-α2 glycoprotein (AZGP1, −1.74-fold, *p* = 6.0 × 10^−3^) which are known for their role in promoting insulin sensitivity. We have also shown the abundance of these proteins in individual samples in Figure 3. 

**Figure 4:**
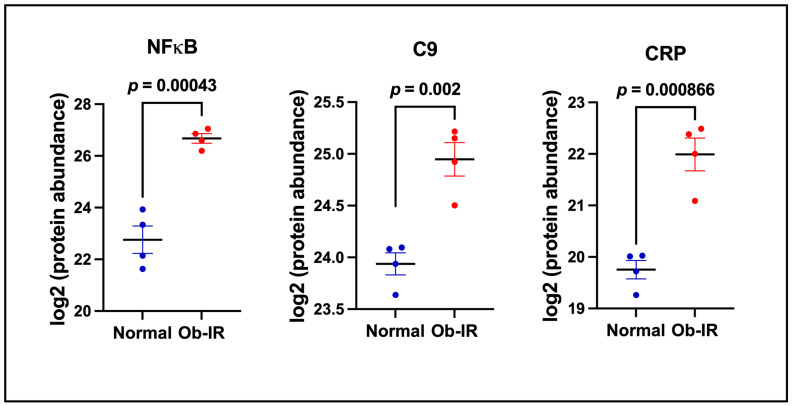
Markers of inflammatory response in exosomes. Proteins altering inflammation in exosomes from normal and Ob-IR subjects. Values are mean ± SEM of 4 samples per group.

Inflammatory pathways play an important role in the development of T2D. Accordingly, we noted an increase in the levels of nuclear factor κB1 (NFκB1, 15-fold, *p* = 4.3 × 10^−4^), C-reactive protein (CRP, 4.4-fold, *p* = 8.66 × 10^−4^), and complement 9 (C9, 2.01-fold, 2.0 × 10^−3^) in EVs from Ob-IR compared to N subjects. As shown in Figure 4, the abundance of these proteins is higher in Ob-IR subjects compared to N subjects. 

**Figure 5:**
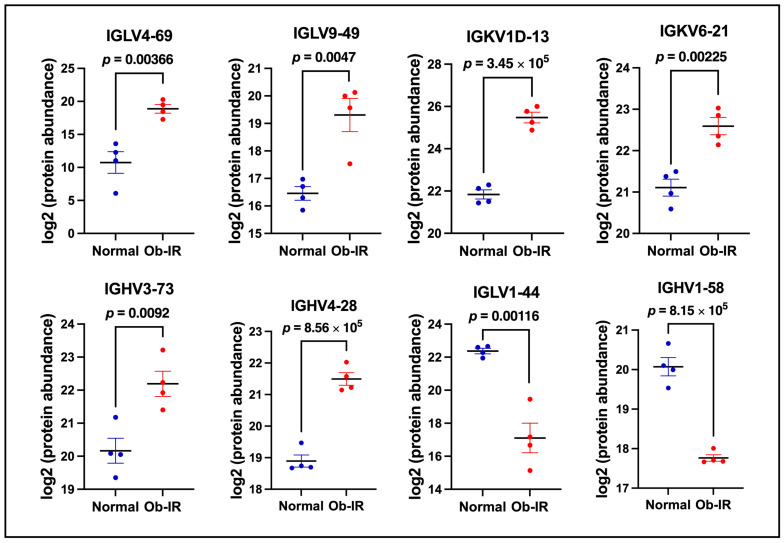
Markers of B cell proliferation/activation. Proteins representing markers of B-cell development and/or activation in exosomes from normal and Ob-IR subjects. Values are mean ± SEM of 4 samples per group.

Intriguingly, markers of immune cell development/activation showed a prominent increase in EVs from Ob-IR individuals compared to controls. In particular, proteins involved in B-cell development/B-cell receptor signaling were significantly higher in Ob-IR subjects compared to N individuals. For example, immunoglobulin lambda variable 4-69 (IGLV4-69) showed a 280-fold increase in Ob-IR subjects compared to the control (*p* = 3.66 × 10^−3^). Moreover, the levels of many other immunoglobulin chains including IGLV9-49 (7.2-fold, *p* = 4.7 × 10^−3^), immunoglobulin kappa variable 1D-13 (IGKV1D-13, 12.4-fold, *p* = 3.45 × 10^−5^), IGKV6-21 (2.8-fold, *p* = 2.25 × 10^−3^), immunoglobulin heavy variable 3-73 (IGHV3-73, 4.1-fold, *p* = 9.2 × 10^−3^) and IGHV4-28 (6.1-fold, *p* = 8.56 × 10^−5^) were significantly upregulated in the Ob-IR group compared to the N group. We also noted a decrease in the levels of some immunoglobulin chains including IGLV1-44 (−38.38-fold, *p* = 1.16 × 10^−3^) and IGHV1-58 (−4.95533-fold, *p* = 8.15 × 10^−5^). The distribution of protein abundance for these markers among different samples in each group is shown in Figure 5. Together, these data show that several markers involved in B-cell development/activation are significantly altered between N and Ob-IR subjects, providing evidence for the role of a novel player in obesity-related IR. 

**Figure 6:**
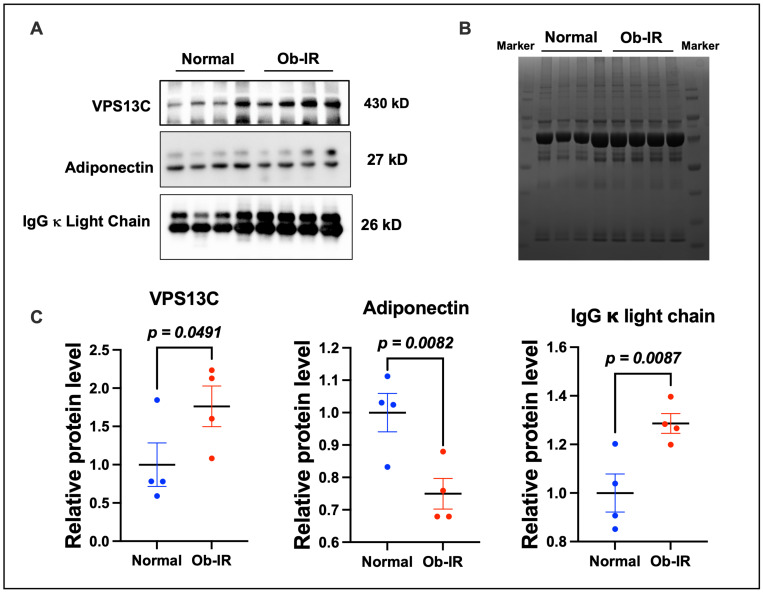
Validation of protein markers. (**A**) Western blot analysis of select proteins confirms the proteomics data. (**B**) Coomassie blue staining of protein bands. (**C**) Densitometric analysis of protein bands normalized to total protein. Values are mean ± SEM of 4 samples per group.

The protein levels of top-altered markers were confirmed by Western blot analyses (Figure 6A). These data show that VPS13C was upregulated in Ob-IR subjects compared to the N group. For B-cell activation, we performed the Western blot analysis for immunoglobulin κ light chain which also showed an upregulation in Ob-IR subjects. In line with our proteomics data, our Western blot analysis showed a decrease in adiponectin in the exosomes derived from Ob-IR subjects compared to N subjects. The proteins detected by Western blot analysis were normalized to total protein in each lane measured by the Coomassie brilliant blue staining of gels (Figure 6B) and the quantification data is shown in Figure 6C. 

### 3.5. Ingenuity Pathway Analysis

Next, all the differential proteins were used for pathway analysis using the Ingenuity Pathway Analysis (IPA) software (version: 111725566). The corresponding Swissport accession numbers were accessed from the human Swiss-Prot database and uploaded to IPA software. The proteins were mapped to disease and function categories and canonical pathways available in IPA databases. The annotated pathways were ranked by the *p*-value. 

**Figure 7:**
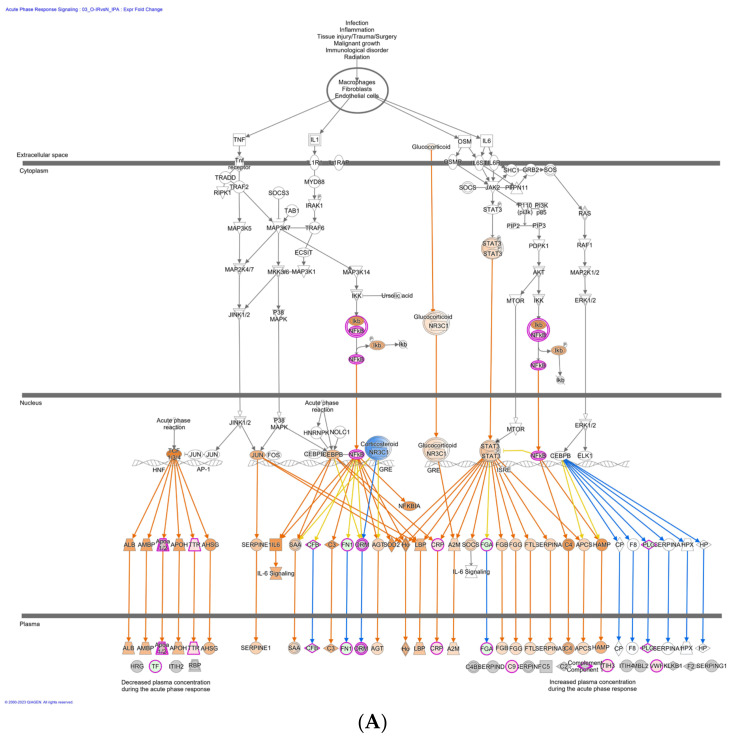

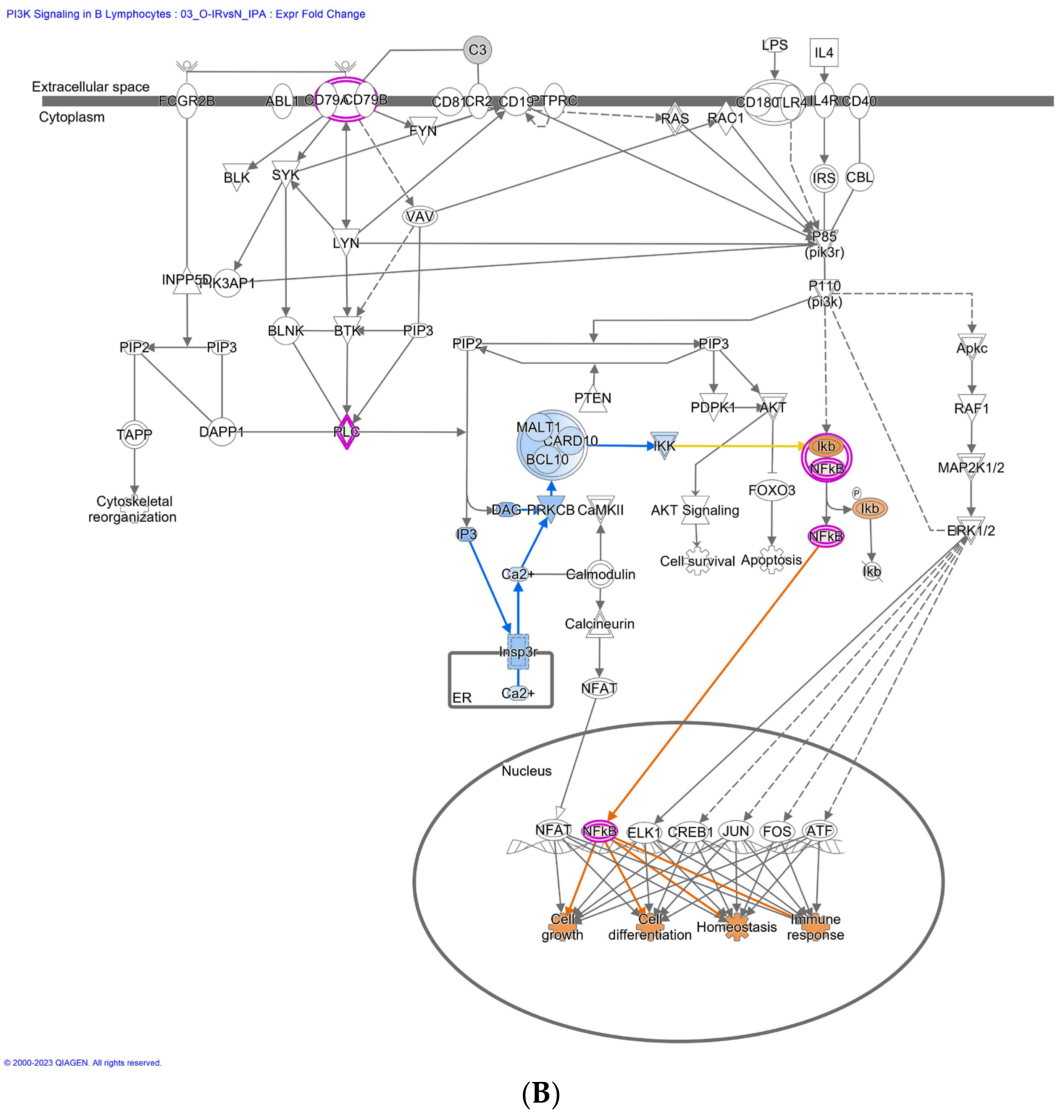
Two of the significantly enriched canonical pathways in Ob-IR group. (**A**) PI3 Kinase signaling in B lymphocytes in Ob-IR group overlapped with DEPs. (**B**) Acute phase reactant signaling in Ob-IR overlapped with DEPs. Red: proteins upregulated in our data set; the intensity of the color indicates the degree of upregulation. The upregulated proteins are highlighted with purple borders. Orange: proteins predicted to be upregulated in our data set; blue: proteins predicted to be downregulated in our data set; and white: proteins not specific to our data set but incorporated as part of the network.

The IPA analysis of DEPs revealed that the significantly affected pathways include the acute phase response signaling (*p* = 1.09 × 10^16^) (Figure 7A) and PI3K signaling in B lymphocytes (*p* = 5.25 × 10^12^) (Figure 7B). The significantly altered canonical pathways include the B-cell development and/or B-cell receptor signaling. The DEPs identified in the top network are associated with humoral immune response, inflammatory response, and hematological system development and function, with a score of 41, indicating a strong association. This is consistent with the GO analysis which also showed that DEPs are enriched mostly in the immune response and immune system process. 

The top upregulated molecules include the DEPs promoting B-cell development/activation (IGLV4-69, IGKV1D-13, IGLV9-49), inflammation (NF-κB), and insulin resistance (VPS13C, TBC1D32). The downregulated molecules include DEPs improving insulin resistance, in particular, ADIPOQ. Together, these data show that B-cell proliferation/activation are altered between N and Ob-IR subjects along with markers of insulin resistance and inflammation. Further, the top-altered proteins, VPS13C, IGKV1D-13, and ADIPOQ can be used as biomarkers to detect high risk individuals or develop therapeutic targets to prevent the development of T2D in Ob-IR subjects.

## 4. Discussion

**Figure 8:**
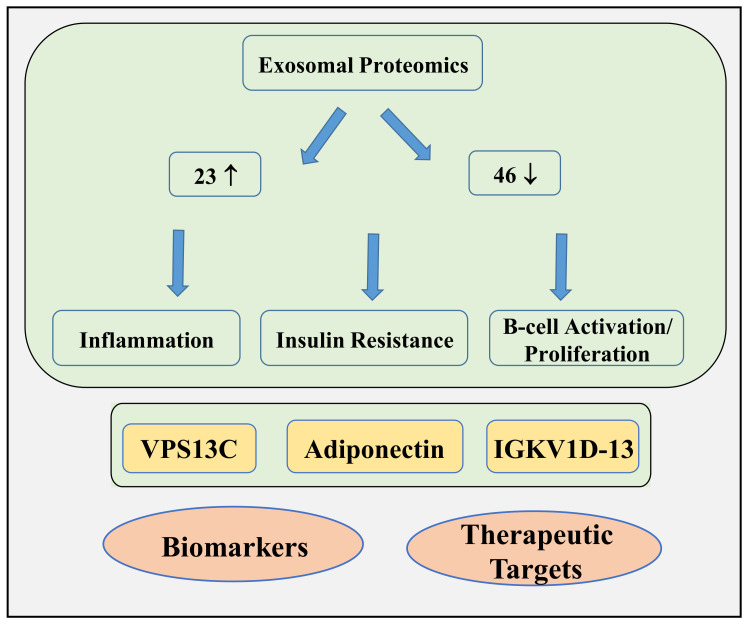
Schematic picture depicting the differentially expressed proteins in exosomes from normal subjects and individuals with obesity and insulin resistance. Proteomics analysis revealed that markers of insulin resistance, inflammation, and B cell activation are increased in exosomes from Ob-IR subjects. The top-altered molecules including VPS13C, adiponectin, and IGKV1D-13 can be considered as biomarkers or therapeutic targets to block the progression of T2D in patients with obesity and insulin resistance.

We have identified 23 upregulated and 46 downregulated proteins in serum exosomes of Ob-IR subjects compared to lean/overweight normal (N) subjects. The DEPs are mainly involved in regulating insulin signaling, inflammation, and B-cell proliferation/activation. GO analysis revealed that the DEPs are mainly located in the extracellular space and regulate immune cell activation. IPA analysis further showed that the top molecules altering between the N and Ob-IR subjects are those regulating insulin signaling, inflammation and B-cell proliferation/activation. Together, our data show that markers of insulin signaling, inflammation, and B-cell activation are altered in the exosomal cargo between N and Ob-IR subjects. These data suggest that exosomal proteins could be used as a biomarker to identify high-risk individuals for T2D and a therapeutic target to prevent T2D in this population (Figure 8). 

Exosomes are increasingly recognized as an important tool to identify biomarkers or understand the pathogenesis of a wide variety of diseases, in particular, obesity-related diseases. With regard to its role as a biomarker, the miRNA cargo of EVs has been frequently studied. For example, a clinical study by Jones et al. (in 2017) showed that circulating extracellular miRNAs secreted by the plasma EVs could be predictive biomarkers for obesity-related IR phenotypes [12]. Another clinical study showed that circulating miRNAs in the exosomes could be a futuristic biomarker for T2D in individuals with obesity [13]. However, very little is known regarding the proteomics profile of exosomes in obesity-related IR. Ge et al. showed that plasma exosomes from subjects with obesity and IR had lower levels of omentin compared to exosomes derived from normal subjects [6]. The top-altered proteins in our study include VPS13C, ADIPOQ, and IGKV1D-13 which can be used as biomarkers for obesity-related IR and to identify individuals with a future risk for T2D. Of note, the VPS13C locus was previously linked to T2D and glycemic traits in a GWAS published earlier [14,15]. Another study has shown that the depletion of VPS13C caused a post-transcriptional increase in the cellular GLUT4 protein and enhanced the cell surface GLUT4 levels in C2C12 myotubes, a process critical for glucose uptake [16]. These reports suggest that a link exists between VPS13C and T2D and our data provide further evidence that VPS13C is increased in serum exosomes from Ob-IR subjects. In addition, our data show that TBC1D32 levels were higher in exosomes from Ob-IR subjects compared to N subjects. Another TBC1D family member, TBC1D1, has been reported to interact with VPS13C and regulate GLUT4-mediated glucose transport in C2C12 myotubes [16]. Our data suggest that TBC1D32 may also have a role in regulating glucose uptake. Indeed, TBC1D32 is known to interact with PPARγ (which plays an important role in regulating glucose uptake in adipocytes) [17]. We also noted a significant increase in TTR, another protein, mediating IR [18]. 

In addition to an increase in the levels of proteins mediating IR, we also noted a decrease in the levels of some proteins which are known to improve insulin sensitivity. As mentioned, we noted a dramatic decrease in adiponectin in exosomes from Ob-IR subjects. Adiponectin is a well-known adipokine, which is considered a biomarker for obesity-related IR [19]. We provide evidence that not only the plasma level, but also the exosome level of adiponectin was lower in subjects with Ob-IR. In addition, the levels of FCN3 and AZGP1, the other proteins improving insulin sensitivity [20,21,22], were lower in exosomes from Ob-IR subjects compared to N subjects. Taken together, the proteomics analysis of serum exosomes revealed markers of IR and future risk of T2D in Ob-IR subjects. 

Although obesity is a strong risk factor for T2D, the mechanisms by which obesity leads to the development of IR remains unclear. Obesity-related inflammation is an important risk factor for the development of IR [23,24]. Accordingly, we noted an increase in inflammatory proteins including NF-κB, CRP, and C9 in Ob-IR subjects. In addition to the inflammatory response, changes in immune cell activation/proliferation which, in turn, alter the immune response, also plays a role in the development of IR. In fact, the most striking finding of our study is that several markers of B cell activation/proliferation were significantly altered between the Ob-IR and N groups. Of note, aberrant B cell activation can result in autoantibody production which, in turn, can promote the development of IR. While type 1 diabetes is an autoimmune disease, accumulating evidence suggests that the activation of B-cells plays a role in the pathogenesis of T2D as well. For example, B cell antibody secretion was higher in patients with obesity and diabetes, compared to patients with obesity and no diabetes [25]. IR in individuals with obesity was associated with a unique profile of IgG autoantibodies [26]. The percentage of B lymphocytes was positively associated with IR, and this was proposed to serve as an appropriate predictor of IR in women with gestational diabetes mellitus [27]. The circulating levels of B2 B cells, a subset of B cells, is positively correlated with hemoglobin A1C in T2D patients [28]. The finding that several markers of antibody fragments were higher in exosomes from Ob-IR subjects, suggests that B cell activation is an important mediator for the development of IR in obesity, and that exosomal B cell activation markers may serve as a therapeutic target to slow down the progression of T2D in obese-IR subjects. 

Our study has many strengths. First, the serum samples from age- and gender-matched subjects from N and Ob-IR groups were used for exosome isolation. Second, we were able to identify several proteins that differentially altered between the N and Ob-IR groups. This could be due to the fact that we used serum samples as opposed to plasma samples which were used in other studies. Finally, we were able to validate the proteomics data by the Western blot analysis which increases the confidence and reliability of the data. Regarding limitations, the sample number is low in our study. However, it is a pilot study, and our future studies will involve a larger number of samples from both male and female subjects. Another limitation of this study and any study assessing the circulating exosomes is that the exosomes preparations include some lipoproteins due to the overlap in their size [29]. Therefore, the changes in DEPs could be partly attributed to the levels of lipoproteins in exosomes. However, the DEPs highlighted in this report do not include any proteins associated with lipoproteins. 

## 5. Conclusions

Taken together, our study suggests that the exosomal proteomics profile is differentially altered between normal subjects and individuals with obesity and IR. These studies highlight the importance of exosomal proteins as biomarkers to identify individuals with a future risk for developing T2D. Our findings are also relevant when considering the targeting of B cell activation to prevent or delay the progression of T2D in high-risk individuals. Future studies are warranted to understand the mechanisms by which these DEP contribute to the pathogenesis of IR in humans.

## Figures and Tables

**Table 1:** Baseline characteristics of study participants.

Measurement	Normal (*n* = 4)	Obese-IR (*n* = 4)	*p* Value
Age (years)	44 ± 4.2	44 ± 6.2	0.9745
Height (in)	98 ± 26.6	71 ± 0.7	0.3337
Weight (lb)	176 ± 29.5	283 ± 15.1 *	0.0179
Body mass index (kg/m^2^)	28 ± 0.6	40 ± 2.4 **	0.0026
Systolic BP (mmHg)	121± 7.5	134 ± 4.7	0.2181
Diastolic BP (mmHg)	77 ± 2.3	88 ± 3.1 *	0.0296
Insulin (µIU/mL)	5 ± 0.4	18 ± 2 ***	0.0008
Glucose (mg/dL)	91 ± 2.7	114 ± 6.5 *	0.0178
HOMA-IR	1 ± 0.1	5 ± 0.6 ***	0.0009
Total cholesterol (mg/dL)	179 ± 9.7	194 ± 11.7	0.3618
Triglycerides (mg/dL)	128 ± 28.1	136 ± 11.4	0.3618
LDL cholesterol (mg/dL)	107 ± 11.1	118 ± 10.6	0.7947
HDL cholesterol (mg/dL)	46 ± 6.3	49 ± 2	0.5003
BUN (mg/dL)	15 ± 0.8	16 ± 2.3	0.6932
Creatinine (mg/dL)	1 ± 0.1	1 ± 0.1	0.5601
Sodium (mmol/L)	139 ± 0.5	139 ± 0.5	0.4881
Potassium (mmol/L)	4 ± 0.1	4 ± 0.1	0.4842
Calcium (mg/dL)	9 ± 0.1	9 ± 0.3	>0.9999
CO2 (mmol/L)	28 ± 0.5	26 ± 1.1	0.1055
Chloride (mmol/L)	103 ± 0.6	104 ± 1.4	0.5334
Alkaline phosphatase (U/L)	59 ± 5.	80 ± 9.5	0.1016
ALT (U/L)	32 ± 2.4	57 ± 10 *	0.0474
AST (U/L)	33 ± 1.8	43 ± 9	0.2940
Bilirubin (mg/dL)	2 ± 0.7	1 ± 0	0.1908

Values are mean ± SEM. BP, blood pressure; HOMA-IR, Homeostatic Model Assessment for Insulin Resistance; ALT, alkaline phosphatase; AST, acid phosphatase. * *p* < 0.05, ** *p* < 0.01, *** *p* < 0.001 vs. normal subjects.

**Table 2:** Differentially expressed proteins in exosomes.

Accession	Gene Names	*p* Value	Log Fold Change (OIR vs. N)	Log2 Protein Abundance (N)	Log2 Protein Abundance (Ob-IR)
Q15848	ADIPOQ	5.14 × 10^−5^	−101.996	20.3359	13.6635
P04114	APOB	0.00264854	−3.43471	27.3783	25.5981
P02655	APOC2	0.00779055	−7.66736	26.2571	23.3183
P02656	APOC3	9.54 × 10^−5^	−5.07365	28.3374	25.9944
P02649	APOE	0.00325172	−4.16134	25.9174	23.8604
P29972	AQP1	0.00884853	−6.3104	18.9832	16.3254
P25311	AZGP1	0.00600575	−1.74242	24.1581	23.3571
P06276	BCHE	0.0030989	−5.01385	22.5725	20.2466
P02747	C1QC	0.00909716	−1.75507	26.6362	25.8247
P07358	C8B	0.00922879	−2.36558	23.9494	22.7072
O43866	CD5L	0.00802754	−4.32778	27.1963	25.0827
P00751	CFB	0.00724882	−3.33718	26.8489	25.1102
Q92496	CFHR4	0.007138	−3.31359	20.048	18.3196
Q9BXR6	CFHR5	0.000347282	−4.41694	17.1679	15.0249
P49747	COMP	0.0101491	−7.1216	17.3908	14.5586
Q96IY4	CPB2	0.000335808	−9.83874	22.2431	18.9446
Q9NQ79	CRTAC1	0.000143608	−7.74126	18.6834	15.7309
P00748	F12	0.000142249	−4.73022	24.3553	22.1134
P12259	F5	0.000641014	−8.68021	21.7898	18.6721
Q15485	FCN2	0.00739225	−3.87146	21.6952	19.7424
O75636	FCN3	0.00974348	−1.8598	24.6062	23.7111
P02671	FGA	0.0034269	−3.49751	23.4074	21.6011
P02751	FN1	0.00461717	−2.20996	26.129	24.985
P69905	HBA2	0.00166529	−2.87522	29.2208	27.6971
P68871	HBB	0.00415203	−3.23454	30.1874	28.4938
A0A0C4DH39	IGHV1-58	8.15 × 10^−5^	−4.95533	20.0733	17.7643
A0A0B4J2H0	IGHV1-69D	3.37 × 10^−5^	−2.08215	21.1561	20.098
P0DP01	IGHV1-8	0.00231204	−3.85637	19.9576	18.0103
A0A0C4DH32	IGHV3-20	0.000622795	−6.30424	21.2174	18.5611
P0DP08	IGHV4-38-2	0.00806797	−2.13237	19.4472	18.3548
P01824	IGHV4-39	0.00806797	−2.13237	23.1349	22.0425
P01834	IGKC	0.00290762	−2.3229	31.4403	30.2244
P01699	IGLV1-44	0.00116193	−38.3801	22.3701	17.1078
P01718	IGLV3-27	0.00911879	−4.65614	21.3846	19.1654
P19652	ORM2	0.00876663	−2.49516	27.8332	26.514
P0DOX7	P0DOX7	0.00290762	−2.3229	30.8636	29.6477
P01833	PIGR	0.00921069	−2.57522	20.6286	19.2639
Q01970	PLCB3	0.000474843	−7.22989	24.0273	21.1733
P00747	PLG	0.000771804	−2.99473	26.7129	25.1304
P55058	PLTP	0.0071463	−4.47115	21.9089	19.7483
P08185	SERPINA6	0.00140644	−14.7621	24.3355	20.4517
Q13103	SPP2	0.00746342	−7.86724	18.2868	15.311
P27105	STOM	0.00238428	−2.2169	19.9259	18.7774
P02787	TF	0.000659321	−2.53408	31.079	29.7375
P02786	TFRC	0.00163401	−4.66298	20.696	18.4747
P07996	THBS1	0.000165745	−35.3192	21.977	16.8346
P02652	APOA2	0.00926537	78.3317	20.577	26.8685
P02748	C9	0.00195931	2.01393	23.9378	24.9478
P15169	CPN1	0.000251491	2.4278	21.0871	22.3667
P22792	CPN2	0.00984944	2.22805	24.357	25.5128
P02741	CRP	0.000866202	4.71278	19.7541	21.9907
Q4L180	FILIP1L	1.32 × 10^−5^	11.5713	20.4921	24.0246
P62805	H4C16	0.00372999	17.8419	13.495	17.6522
A0A0B4J1V6	IGHV3-73	0.00917639	4.07111	20.1666	22.192
A0A0C4DH34	IGHV4-28	8.56 × 10^−5^	6.05381	18.8953	21.4932
A0A0B4J2D9	IGKV1D-13	3.45 × 10^−5^	12.4358	21.8398	25.4763
A0A0C4DH24	IGKV6-21	0.00225202	2.80145	21.107	22.5931
P01706	IGLV2-11	0.00978276	2.39056	22.1182	23.3756
A0A075B6H9	IGLV4-69	0.00365724	280.396	10.7438	18.8752
A0A0B4J1Y8	IGLV9-49	0.00467814	7.21009	16.4558	19.3058
Q06033	ITIH3	0.00727725	2.96715	25.4247	26.9938
P19838	NFKB1	0.000426808	15.1041	22.7597	26.6766
O60313	OPA1	2.83 × 10^−5^	40.72	13.8771	19.2248
P22891	PROZ	0.00859579	2.50669	17.3095	18.6352
P20742	PZP	0.00298606	2.1067	23.7413	24.8163
Q96NH3	TBC1D32	0.000483639	17.7464	14.9629	19.1124
P02766	TTR	0.00174771	1.90673	27.9756	28.9067
Q709C8	VPS13C	0.004229	152.354	15.4026	22.6539
P04275	VWF	0.00300269	2.78745	20.2546	21.7336

## Data Availability

The proteomics data presented in the study are openly available in [MassIVE Repository (MSV000092982)]. The other raw data supporting the conclusions of this article will be made available by the authors on request.
